# Cardiovascular disease risk and secondary prevention of cardiovascular disease among patients with low health literacy

**DOI:** 10.1007/s12471-017-0963-6

**Published:** 2017-02-28

**Authors:** T. M. van Schaik, H. T. Jørstad, T. B. Twickler, R. J. G. Peters, J. P. G. Tijssen, M. L. Essink-Bot, M. P. Fransen

**Affiliations:** 1 Department of Cardiology, Academic Medical Centre, University of Amsterdam, Amsterdam, The Netherlands; 2 Department of Public Health, Academic Medical Centre, University of Amsterdam, Amsterdam, The Netherlands; 3 Department of Endocrinology, Diabetology and Metabolic Diseases, University Hospital, Antwerp, Belgium; 4 Department of Endocrinology, Diabetology and Metabolic Diseases, AZ Monica Hospital, Deurne/Antwerp, Belgium

**Keywords:** Health literacy, Coronary artery disease, Cardiovascular disease risk, Secondary prevention, Nurse coordinated prevention program

## Abstract

**Objective:**

To explore the association between health literacy and the risk of cardiovascular disease (CVD), and to assess the differential effects by health literacy level of a nurse-coordinated secondary prevention program (NCPP) in patients with coronary artery disease (CAD).

**Methods:**

Data were collected in two medical centres participating in the RESPONSE trial (Randomised Evaluation of Secondary Prevention by Outpatient Nurse SpEcialists). CVD risk profiles were assessed at baseline and 12-month follow-up using the Systematic Coronary Risk Evaluation (SCORE). Health literacy was assessed by the short Rapid Estimate of Adult Literacy in Medicine (REALM-D) and the Newest Vital Sign (NVS-D); self-reported health literacy was evaluated by the Set of Brief Screening Questions (SBSQ-D).

**Results:**

Among 201 CAD patients, 18% exhibited reading difficulties, 52% had difficulty understanding and applying written information, and 5% scored low on self-reported health literacy. Patients with low NVS-D scores had a higher CVD risk [mean SCORE 5.2 (SD 4.8) versus 3.3 (SD 4.1), *p* < 0.01]. Nurse-coordinated care seemed to reduce CVD risk irrespective of health literacy levels without significant differences.

**Conclusion:**

Inadequate health literacy is prevalent in CAD patients in the Netherlands, and is associated with less favourable CVD risk profiles. Where many other forms of CVD prevention fail, nurse-coordinated care seems to be effective among patients with inadequate health literacy.

## Introduction

Patients with manifest coronary artery disease (CAD) are at high risk of recurrent coronary events and death. Secondary prevention, consisting of a healthy lifestyle and optimal drug therapy, can reduce this risk [1]. However, the use of such evidence-based secondary prevention is far from optimal [2]. It is unknown whether specialised secondary prevention strategies as recommended by the current guidelines (i. e. multidisciplinary cardiac rehabilitation, preventive programs for therapy optimisation, adherence and risk factor management, and nurse and allied health professional led programs) are effective for all CAD patients, in particular those with low health literacy [1]. Health literacy refers to individual skills to obtain, process, and understand basic health information and services needed to make appropriate health decisions [3, 4]. Lower health literacy is associated with less well controlled blood pressure in primary care patients with hypertension and heart disease [5] and worse adherence to cardiovascular preventive drugs [6]. Interventions tailored to low health literacy appeared effective in improving medication adherence [7, 8].

It is estimated that almost 27% of the Dutch population has limited health literacy, but the prevalence of low health literacy in CAD patients in the Netherlands is unknown [9]. Studies in the United States reported low levels of health literacy in 27 to 54% of patients with heart failure [10–12]. Health literacy is associated with lower educational level, non-Western ethnic background and age [9, 13, 14]. Reading, listening, and calculating skills, important components of health literacy, are considered to be a mediator in the association between educational level and cardiovascular risk [15, 16]. Data on the prevalence and consequences of low health literacy in CAD patients in Europe are limited and evidence on the effect cardiovascular preventive care or interventions in low health literacy CAD patients is lacking [17–20]. The Dutch RESPONSE trial (Randomised Evaluation of Secondary Prevention by Outpatient Nurse SpEcialists) showed that patients randomised to a nurse-coordinated prevention intervention had better control of risk factors and a predicted relative risk of mortality than the control group. The outcome was measured by SCORE, a risk assessment tool based on age, gender, smoking status, systolic blood pressure, and cholesterol levels [21, 22]. This scheduled, individual, face-to-face guidance could potentially be effective among patients with inadequate health literacy, since it enables tailoring of information and support to their lower ability to apply information on, for example, lifestyle and medication in their daily life. We therefore expect that especially patients with low health literacy would benefit from a nurse-coordinated intervention.

The general aim of this study was to gain insight into the prevalence of health literacy among patients with established CAD in the Netherlands, and to investigate the effectiveness of nurse-coordinated secondary prevention on CVD risk in patients with low and adequate health literacy. For this purpose we used SCORE, an assessment tool for CVD risk that is based on age, gender, smoking status, systolic blood pressure, and cholesterol levels.

Our research questions were:

1. What is the prevalence of inadequate health literacy among patients with established CAD in the Netherlands?
2. What is the association between inadequate health literacy and cardiovascular risk profiles (as assessed using SCORE)?
3. Is there a difference in effectiveness of nurse-coordinated care in patients with inadequate and adequate health literacy?

## Methods

### Research population and recruitment

We performed a cross-sectional survey embedded in the RESPONSE trial, a multicentre randomised, clinical trial in the Netherlands that investigated the effect of a nurse-coordinated prevention program (NCPP) on top of usual care (controls) [21, 22]. The protocol of the RESPONSE trial was approved by the institutional committees on human research in all recruiting hospitals. The current study was approved as an addendum to the main trial by the institutional committee on human research of the Academic Medical Center – University of Amsterdam, Amsterdam, the Netherlands.

Referral to the NCPP included up to four visits during the first six months after inclusion. At each visit, patients were seen by a trained nurse specialist. The NCPP focused on [1] promoting healthy lifestyles, [2] managing biometric risk factors and [3] increasing medication adherence.

Patients aged 18–80 years were eligible for participation in RESPONSE if they had been hospitalised for an acute coronary syndrome (ST-segment elevation myocardial infarction (STEMI), non-ST-segment elevation myocardial infarction (non-STEMI), or unstable angina). Exclusion criteria were: visits to the prevention program not feasible; not available for follow-up; insufficient mastery of Dutch; surgery, percutaneous coronary intervention or other interventions expected within 8 weeks after index event; limited life expectancy; previously enrolled in an NCPP; NYHA class 3 or 4 congestive heart failure.

Participants for our survey were recruited between February and June 2010 in two participating centres of the RESPONSE trial (Academic Medical Centre Amsterdam and Medical Spectrum Twente Enschede). All RESPONSE participants received an introductory letter describing the objective of the current study and data collection on health literacy, and were subsequently invited for the survey by telephone.

### Data collection

During the RESPONSE trial, data were collected at baseline and at 12-month follow-up by patient files and patient interviews, as appropriate. Detailed information on data collection in RESPONSE has been reported by Jørstad et al. [21, 22].

Background characteristics consisted of: gender; educational level (classified as low, medium or high); ethnic background (patients’ and his/her parents’ country of birth); weight; height; cardiovascular history; index event (acute coronary syndrome) and any revascularisation; smoking status prior to index event.

*Cardiovascular risk* was assessed using SCORE, which estimates the absolute 10 years cardiovascular mortality risk based on age, gender, total cholesterol, systolic blood pressure and smoking status [23]. Blood pressure was measured using a validated automated sphygmomanometer. Blood samples were analysed by the local laboratories for the measurements of lipid profiles, including low-density lipoprotein cholesterol. Patients were instructed to observe an 8‑hour period of fasting prior to blood sampling.

*Health literacy* was assessed in separate personal interviews at or after 12 months of follow-up in RESPONSE. Interviews were performed at the Academic Medical Centre Amsterdam or by telephone. We used the following instruments to measure health literacy: The Rapid Estimate of Adult Literacy in Medicine (REALM) is a word recognition test consisting of 66 health-related words divided into three lists of increasing complexity. Examples are cancer (list 1), hormones (list 2), hypertension (list 3) [24]. Respondents receive one point if they pronounce a word correctly. This results in a total score range of 0–66, which is converted to a US school grade estimate of reading ability. Scores below 18 indicate that patients might not be able to read most low literacy materials, scores between 19 and 44 indicate that patients need low literacy materials, scores between 45 and 60 indicate that patients may have problems in reading most patient education materials, and scores above 60 indicate that patients are probably able to read most patient education materials [24].

The Newest Vital Sign (NVS) includes objective assessments of numeracy and the ability to understand and apply written information. It consists of six questions about the information on a food label (e. g. If you eat the entire container, how many calories will you eat?), resulting in a total score range of 0–6. A score between 0 and 1 suggests a likelihood of ≥50% of limited literacy, 2–3 indicates the possibility of limited literacy, and 4–6 almost always indicates adequate literacy [25].

The Set of Brief Screening Questions (SBSQ) measures perceived health literacy [26, 27]. It consists of three statements about the patient’s perceived ability to understand and apply health information. Responses are scored on a 5-point Likert scale from 0 to 4, added up and averaged. The response of ‘somewhat’ or less provided optimum sensitivity and specificity and is considered as an optimal screening threshold in most studies [26, 27]. This means that an average score of ≤2 indicates inadequate health literacy, a score >2 indicates adequate health literacy.

We previously translated these measures into Dutch (REALM-D-D, NVS-D-D, SBSQ-D-D), assessed the psychometric properties and evaluated the cross-cultural applicability of the measures [28–30]. The Cronbach’s alpha coefficient for the Dutch REALM-D was 0.91. Cronbach’s alpha was 0.78 for the Dutch NVS-D. Both coefficients are regarded as sufficient for group comparisons. The Cronbach’s alpha coefficient for the Dutch SBSQ-D was 0.69, which indicates an acceptable internal consistency. All three measures were able to significantly (*p* ≤ 0.01) differentiate between low and high educated patients on the basis of statistically significant differences in mean scores. The correlation of REALM-D scores was strongest with the SBSQ-D scores (r = 0.59, *p* = 0.00). The correlation between REALM-D and NVS-D was moderate (r = 0.32, *p* = 0.00/r = 0.22, *p* = 0.04). For the REALM-D and NVS-D, patients received printed forms by regular mail, in a sealed envelope, labelled with instructions not to open the envelope until the start of the interview. This was done to ensure that the patient did not study the health literacy tests in advance.

## Statistical methods

Health literacy scores were dichotomised into adequate and inadequate health literacy per measure, following predefined cut-off points [26, 27, 29, 30]. For REALM-D a score <60 indicated inadequate health literacy, for NVS-D this was <2, and for SBSQ-D this was <3. Patients with missing biometric values to calculate SCORE at baseline (2%) or 12-month follow-up (3%) were excluded from our analyses. Descriptive analyses were used to calculate the prevalence of inadequate health literacy among CAD patients in the Netherlands (RQ1). The association between health literacy and SCORE (RQ2) was analysed by two sample t‑tests. Since educational level, ethnic background, and age are generally associated with both health literacy as well as cardiovascular risk [9, 13, 14, 31, 32], we corrected for these variables in our analyses. We performed stepwise linear regression analyses to correct for educational level, ethnic background and age in the association between REALM-D and score, NVS-D and score, and SBSQ-D and SCORE. We used the two-sample t‑test to assess differences in the effect of nurse-coordinated care between health literacy groups (RQ3). We first analysed the mean change in SCORE between baseline and 12-month follow-up for the intervention and control group for the total population. We then performed the same analyses for strata of low and adequate health literacy; *p*-values of ≤0.05 were considered significant. We used SPSS statistics V.23 for all statistical analyses.

## Results

### Response and patient characteristics

**Fig. 1:**
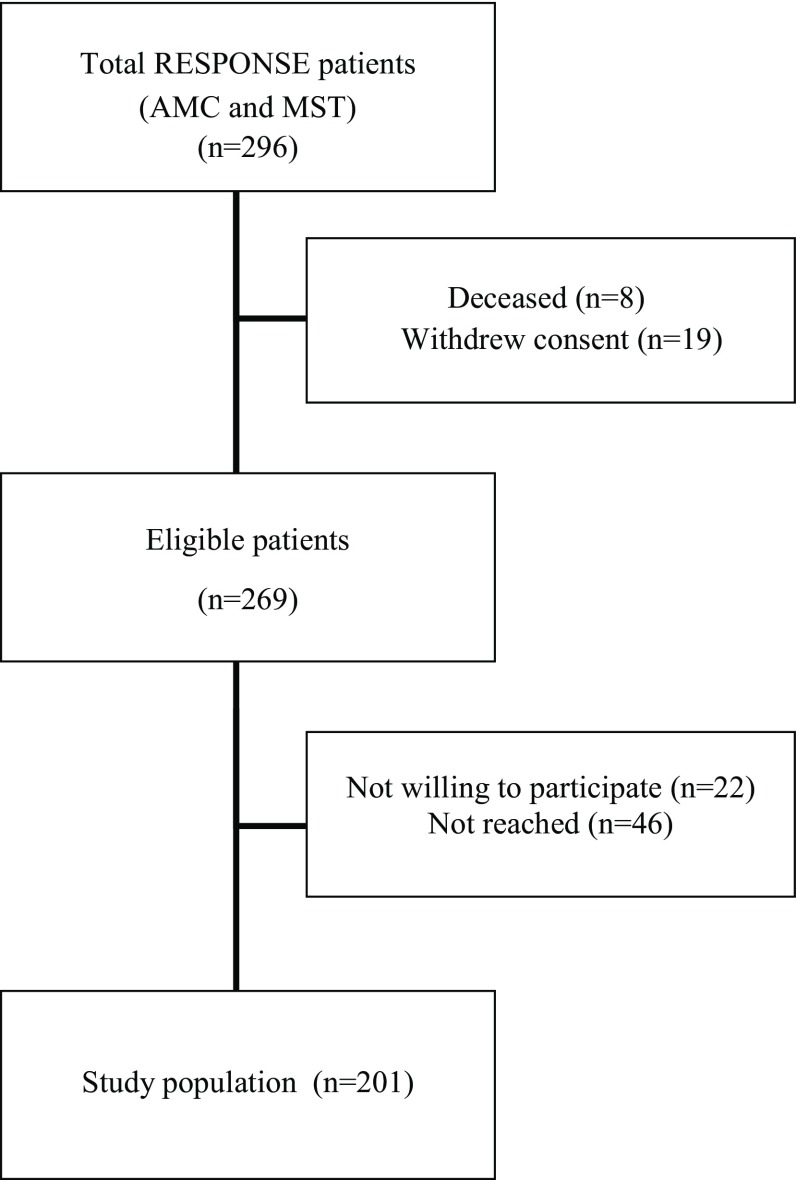
Flowchart showing the response, *AMC* Academic Medical Center, *MST* Medisch Spectrum Twente

Fig. 1 presents the study flowchart. In total, 269 of 296 patients were eligible to participate (8 patients died and 19 patients withdrew consent before the start of the current study). Of these 269 patients, 22 patients refused participation due to lack of time (*n* = 16), fatigue (*n* = 4), or family circumstances (*n* = 2), and 46 patients could not be reached. In total, 201 patients were included in our study (75%). Patient characteristics are shown in Table 1. Mean age was 56 years, and 80% were male, 84% had a Dutch ethnic background. At baseline, 52% were diagnosed with STEMI, 30% had non-STEMI, and 19% were diagnosed with unstable angina. In total, 17% of all patients had a history of prior myocardial infarction, while the majority of patients had no history of cardiovascular disease (73%).

**Table 1:** Background characteristics study population (*n* = 201)

	*n*	(%)	Mean (SD)
*Demographics*			
Age, mean age in years (SD)			56 (9.3)
*Educational level*			
Low (primary)	24	(12)	–
Middle (secondary and/or tertiary)	128	(64)	–
High (quaternary)	48	(24)	–
Ethnic background (Dutch)	169	(84)	–
*Index event*			
ST-segment elevation myocardial infarction	103	(52)	–
Non ST-segment elevation myocardial infarction	61	(30)	–
Unstable angina	37	(18)	–
History of myocardial infarction	34	(17)	–
History of stroke	11	(6)	–
History of peripheral artery disease	11	(6)	–
History of congestive heart failure	2	(1)	–
No history of cardiovascular disease	146	(73)	–
*Cardiovascular risk*			
SCORE			4.2 (4.5)
Hypertension	85	(42)	–
Dyslipidaemia	149	(74)	–
Smoking	95	(47)	–
Diabetes	33	(16)	–
Family history of cardiovascular disease	129	(64)	–
*Interventions at index event*			
Percutaneous coronary intervention	161	(80)	–
Coronary artery bypass graft	14	(7)	–
No revascularisation	31	(15)	–
NCPP group	94	(47)	–

*NCPP* Nurse Coordinated Prevention Programme, *SCORE* Systematic COronary Risk Evaluation

4 missing observations calculating SCORE, 3 missing observations in NCPP

### Prevalence of inadequate health literacy

Table 2 presents the number of patients with low or adequate health literacy. According to the REALM-D, 34 patients (18%) had inadequate reading skills, while the NVS-D showed that 103 patients (52%) had difficulty understanding and applying written information. Eleven patients (5%) reported having difficulties in understanding and applying health information (SBSQ-D).

**Table 2:** Health literacy levels and association between health literacy (HL) and cardiovascular risk (*n* = 197)

		Health literacy *n* (%)		Mean SCORE (SD)	Mean difference in SCORE between HL groups (*P*; adjusted *P* ^a^)
NVS-D	Adequate HL	96	(48)	3.28 (4.1)	−1.88 (<0.01; <0.01)
	Inadequate HL	103	(52)	5.16 (4.8)	–
REALM-D	Adequate HL	164	(82)	4.11 (4.6)	−0.71 (0.41; 0.21)
	Inadequate HL	34	(18)	4.82 (4.4)	–
SBSQ-D	Adequate HL	189	(95)	4.08 (4.4)	−2.42 (0.08; 0.04)
	Inadequate HL	11	(5)	6.50 (6.7)	–

^a^Adjusted for educational level and ethnic background. 3 missing observations on REALM-D; 2 missing observations on NVS-D; 1 missing observation on SBS

### Association between health literacy and CVD risk profiles

Table 2 further presents SCORE at baseline (mean; SD) stratified by the level of health literacy. SCORE was higher in patients with inadequate health literacy as compared with those with adequate health literacy, according to the NVS-D, the REALM-D and the SBSQ-D. Patients with low NVS-D scores had a higher CVD risk (mean SCORE 5.2 (SD 4.8) versus 3.3 (SD 4.1), *p* < 0.01). This difference remained significant after correction for educational level and ethnic background. After correction for educational level, ethnic background and age, the difference in SCORE was no longer significant. The difference in SCORE for the SBSQ-D was significant when the model was corrected for educational level and ethnic background (*p* = 0.04) but not in the other models. The difference in SCORE for REALM-D was not significant in any regression model.

### Association between health literacy and effectiveness secondary prevention

Table 3 shows the mean change in SCORE after attending the NCPP for the intervention and control group (12 months of follow-up relative to baseline), stratified by health literacy level. We did not find significant differences in mean change between the intervention and control group for the total population (*p* = 0.22) or between patients with inadequate and adequate health literacy (*p* = 0.23). Patients with inadequate health literacy in the intervention group showed a greater improvement in SCORE than patients with adequate health literacy. For example, patients who had low health literacy according to the REALM-D had a change in SCORE of −0.96, this change was −0.28 in those that had adequate health literacy. While patients with adequate health literacy in the control group improved in SCORE after 12 months of follow-up, those with inadequate health literacy did not improve. However the observed differences between health literacy groups were statistically not significant.

**Table 3:** Mean change in SCORE at 12 months follow-up among intervention and control group, stratified by health literacy (HL) level (*n* = 201)

		Intervention group (*n* = 94)		Control group (*n* = 107)		Difference intervention and control	
		Mean change in score between baseline and follow-up	*P*	Mean change in score between baseline and follow-up	*P*	Difference in mean change	*P*
Total		−0.38	(0.13)	−0.07	(0.78)	0.31	0.22
NVS-D	Adequate HL	−0.38	(0.28)	−0.14	(0.60)	0.24	0.73
	Inadequate HL	−0.41	(0.29)	−0.01	(0.98)	–	0.23
REALM-D	Adequate HL	−0.28	(0.31)	−0.15	(0.53)	0.13	0.57
	Inadequate HL	−0.96	(0.14)	+0.29	(0.70)	–	0.48
SBSQ-D	Adequate HL	−0.40	(0.14)	−0.13	(0.57)	0.27	0.44
	Inadequate HL	−0.01	(0.86)	+1.22	(0.60)	–	0.53

4 missing observations calculating Systematic Coronary Risk Evaluation (SCORE); 3 missing observations on REALM-D; 2 missing observations on NVS-D; 1 missing observation on SBS-Q

## Discussion

Our study shows that inadequate health literacy is highly prevalent in patients with CAD, ranging from 18% who have inadequate reading skills to 52% who have difficulty understanding and applying written information. Patients with low health literacy had significantly worse CVD risk profiles. However, the NCPP led to similar reductions in CVD risk both in individuals with inadequate and adequate health literacy and was thus equally effective for all.

Health literacy scores found in our study are comparable with health literacy levels in the general population in the Netherlands, the UK and Ireland [28–30, 33, 34]. A limited number of studies have investigated the association between health literacy and CVD risk. These studies were either performed in a general population (primary prevention) or assessed independent risk factors instead of integrated risk profiles. Martin et al. showed that inadequate literacy skills were associated with higher CVD risk as measured by the Framingham algorithm in the general population [16]. This association was only statistically significant in women. However, this study was performed in a markedly different population in the US, consisting of young individuals in their mid-forties without previous CVD. McNaugton et al. found that low health literacy (REALM-D) was independently associated with uncontrolled blood pressure among 423 urban, primary care patients with hypertension and coronary disease [5]. Aranha et al. found no association between health literacy and independent CVD risk factors among 150 elderly patients seeking care at a patient-centred medical home in the US [35].

To our knowledge, our study is the first to investigate the impact of health literacy on the effects of secondary prevention by nurse coordinated care as prescribed in the current European guidelines [1]. We observed that patients with inadequate health literacy in the intervention group SCORE had improved risk profiles at 12-month follow-up, while those in the control group showed no improvement. Although this difference was not significant, it suggests the specific need for an NCPP among CAD patients with low health literacy. This is in line with studies in the US which demonstrated that patients with low health literacy and heart failure have a stronger preference for patient-centred information, and that they benefit more from self-management programs using adjusted educational materials and scheduled (telephone) follow-up [36, 37].

Several factors need to be taken into account when interpreting our results. First, we did not find any statistically significant results regarding the effectiveness of the NCPP. This is in contrast to the findings in the RESPONSE trial where good risk factor control was achieved in 35% of patients in the intervention group compared with 25% in the control group at 12 months (*p* = 0.003). This difference is probably related to the fact that our sample was much smaller (201 compared with 754) than the sample in the RESPONSE trial, and that we only recruited in two medical centres that participated in the RESPONSE trial. A larger sample is needed to confirm the significance of the differences in the effectiveness that we found between health literacy groups.

Second, all patients participating in clinical trials are able to read and provide written informed consent, potentially leading to an oversampling of literate patients. To account for low literacy, we approached patients in person or by telephone. Lower ability to read the introductory letter was therefore not necessarily a limitation. However, patients choosing to participate in randomised clinical trials are not representative of the general patient population. Furthermore, data (for example on health literacy) were lacking on deceased patients and patients who withdrew consent. Third, the absolute estimates of the SCORE function are inaccurate in secondary prevention. We were unable to use the SMART score for secondary prevention [30], since C‑reactive protein and kidney function were not assessed in the RESPONSE trial. However, the difference in SCORE between the two groups provides an estimate of the relative overall impact of a risk factor intervention.

## Conclusion

Inadequate health literacy is highly prevalent in patients with documented CAD, and is associated with adverse risk profiles. It seems that an NCPP leads to the improvement of risk profiles and that this does not differ between patients with inadequate and adequate health literacy. A larger sample is needed to confirm the significance of the differences in the effectiveness that we found between health literacy groups.

## Implications

Patients with inadequate health literacy are generally less likely to receive and/or follow preventive treatment. However, because of their less favourable CVD risk profile, their need for effective secondary prevention is greater. We found that an NCPP is equally effective across health literacy levels. Where many other forms of prevention fail, an NCPP seems effective among patients with inadequate health literacy and therefore offers a promising concept of secondary prevention of CVD.

The protocol for our survey was approved by the institutional committees on human research of both participating hospitals.
